# Cisheteronormativity, Conversion Therapy, and Identity Among Sexual and Gender Minority People: A Narrative Inquiry and Creative Non-fiction

**DOI:** 10.1177/10497323221126536

**Published:** 2022-09-18

**Authors:** David J. Kinitz, Travis Salway

**Affiliations:** 1 Social and Behavioural Health Sciences Division, Dalla Lana School of Public Health, 7938 University of Toronto, Toronto, ON, Canada; 2 Faculty of Health Sciences, 1763 Simon Fraser University, Burnaby, BC, Canada; 3 British Columbia Centre for Disease Control, Vancouver, BC, Canada; 4 Centre for Gender and Sexual Health Equity, Vancouver, BC, Canada

**Keywords:** identity,, conversion therapy,, lesbian,, gay,, bisexual,, transgender,, queer,, narrative inquiry,, master narrative framework,, creative non-fiction,, sexual and gender minority health

## Abstract

Sexual and gender minorities (SGMs) navigate systems of oppression that reify cisgender and heterosexual norms (cisheteronormativity) while developing their identities. ‘Conversion therapy’ represents a particularly prominent and harmful threat in this landscape. We explore how SGM who experienced conversion therapy develop their identities to understand antecedents to mental health struggles in this population. In-depth interviews were conducted with 22 people in Canada. A ‘master narratives’ framework combined with Polkinghorne’s narrative analysis were used to explore individual-structural relations that affect identity in settings where cisheteronormative master narratives are amplified (i.e., conversion therapy). We present research findings through a creative non-fiction, which includes learning cisheteronormative master narratives; internalizing master narratives; feeling broken and searching for alternatives; and embracing self-love amidst pain. The amplification of master narratives through conversion therapy leads to conflict and delays in adopting a coherent identity. Health professionals should enact institutional practices that affirm SGM and thereby deemphasize cisheteronormativity.

## Conversion Therapy and Sexual Orientation and Gender Identity and Expression Change Efforts

Sexual and gender minority (SGM) people experience structural systems of oppression that reify cisgender and heterosexual norms as the dominant status quo (i.e., cisheteronormativity) throughout their lives (Chevrette & Eguchi, 2020). Amplified assertions of cisheteronormativity are experienced by SGM through practices of conversion therapy and other sexual orientation and gender identity and expression change efforts (SOGIECE; Salway et al., 2021; The Trans PULSE Canada Team, 2019). SOGIECE include practices that attempt to deny, change, or suppress sexual orientations and gender identities that are not heterosexual or cisgender (Power et al., 2022). Conversion therapy is one of the most formalized and explicit forms of SOGIECE, a more encompassing acronym used throughout this article; both are change efforts, part of a larger system of efforts to discourage SGM identities and behaviours, regardless of whether any intrinsic ‘change’ is intended (Kinitz et al., 2022). Conversion therapy has been called the ‘sharpest edge’ of cisheteronormativity and promotes developing stronger non-romantic and non-sexual relationships with those of the same gender, enacting stereotypical traits of the gender/sex one is assigned at birth, and engaging in psychotherapy to reduce sexual attraction toward the same gender to strengthen attraction to the ‘opposite’ gender, among other cisheteronormative expectations (CBRC, 2020). Approaches to SOGIECE are diverse and are practiced by a range of professionals, including trained health practitioners, religious leaders, and members of families and communities (Bishop, 2019; Kinitz et al., 2022; Power et al., 2022).

It can be estimated that millions of SGM across the globe, in higher and lower-middle income countries, continue to be exposed to these marginally regulated practices occurring in secular and religious settings (Bishop, 2019). Prevalence of SOGIECE differ by country and can vary widely depending on definitions of SOGIECE and participant demographics (e.g., age, gender modality). For example, one study in the US reported 34% of SGM were subjected to SOGIECE (Ryan et al., 2020), whereas a study among SGM Australian youth reported 4% exposure to conversion practices (Jones et al., 2021). Further, a Canadian study reported that up to 21% of sexual minority men experienced some form of change effort (i.e., SOGIECE) and 10% experienced conversion therapy (Salway et al., 2021). Studies focussed on trans people specifically found prevalence rates of 14% in the US and 11% in Canada (The Trans PULSE Canada Team, 2019; Turban et al., 2019).

Qualitative studies suggest that SOGIECE look similar across Western countries and can be traumatic, impacting various aspects of one’s mental health (Goodyear et al., 2022; UK Government Equities Office, 2021). A growing evidence-base demonstrates that SOGIECE are associated with increased rates of anxiety, depression, isolation, and suicidal ideation and attempts (Blosnich et al., 2020; Jones et al., 2021; Mallory et al., 2019; Salway et al., 2020; Turban et al., 2019). A recent Australian study highlights that despite SGM youth already experiencing increased rates of suicide and poor mental health, SGM youth who experienced conversion therapy were almost four times more likely to attempt suicide than SGM youth who did not experience conversion therapy (Jones et al., 2021). Similarly, a large US study among cisgender sexual minority adults found suicidality doubled among those who experienced sexual orientation change efforts (Blosnich et al., 2020). Given the profound harms of these practices, large, national studies are taking place globally to address contemporary gaps in the provision of social policy and health and social supports for survivors (Jones et al., 2021; Mallory et al., 2019; Salway et al., 2021). Furthermore, policy arenas addressing SOGIECE are contentious and require qualitative health research to provide nuance and highly useful context amidst a growing quantitative literature.

## Sexual and Gender Minority People’s Identity

Few studies have examined the impacts of SOGIECE on identity, despite the known and significant connections between one’s sense of identity, mental health, and wellbeing (Adler, 2012; Fingerhut et al., 2010). We conceptualize identity based on Hammack’s (2008) definition that identity is ‘…ideology cognized through the individual engagement with discourse, made manifest in a personal narrative constructed and reconstructed across the life course and scripted in and through social interaction and social practice’ (p. 230). Identity construction is a critical process of the life course that achieves a consistent feeling of personal cohesion and is particularly important throughout adolescence and young adulthood (McAdams & Zapata-Gietl, 2015). It can significantly impact how one progresses through life milestones, particularly given one’s situatedness to dominant cultural contexts (McAdams & Zapata-Gietl, 2015; Syed & McLean, 2016).

Sexual and gender identity is a substantial aspect of one’s narrative and brings with it unique challenges, particularly for many SGM (e.g., keeping aspects of one’s life secret or feeling isolated). Cisgender heterosexual people do not undergo the same sexual and gender identity development processes as SGM, given that their sexuality and gender identities are presumed the default (Hammack, 2018). Conversely, sexual and gender identity construction among SGM can occupy much of their lives as they navigate cisheteronormative narratives. Experiences of discrimination and marginalization directly impact one’s ability to develop their personal identity, beyond sexual and gender identity, in healthy and positive ways (McLean et al., 2018). The widespread marginalization of SGM identities and behaviours learned in early life, combined with day-to-day experiences of discrimination throughout SGM adolescence and adulthood, result in years of having to hide aspects of identity, avoid social interactions with those who do not signal pro-SGM stances, internalize self-doubt, among many other challenges in constructing a personal narrative and secure identity (Hammack, 2005). This marginalization and years of navigating stigma has direct impacts on SGM health (Brooks, 1981; Pachankis et al., 2020).

Research regarding sexually diverse identity development is expansive, with a small but growing corpus of research focused on gender diverse identity development (Bradford & Syed, 2019; Hammack, 2018). However, much of the literature pays insufficient attention to the broader sociocultural contexts in which personal identities are constructed (Syed & McLean, 2021). SOGIECE is one such context. Through the lens of SOGIECE, SGM identities are often considered to be disordered. Elizabeth Moberly, a psychologist credited with introducing the Christian-informed ideologies that led to the reuptake of SOGIECE in the 1980s, believed that homosexuality could be ‘cured’ (Johnston & Jenkins, 2006). People who experience SOGIECE are often desperate, lured by claims of being ‘healed’ or ‘cured’, have feelings of self-loathing exacerbated, and are often blamed and left feeling as though they have personally failed when they are not ‘cured’ (Johnston & Jenkins, 2006; Kinitz et al., 2022). Thus, SOGIECE is not only detrimental to the personal narratives and identities of SGM people (i.e., through suppression and denial of SGM identities) but a host of other identity-related issues.

To date, there has been limited interrogation of the mechanisms by which SOGIECE contribute to particular mental health outcomes. Past theorizing connects personal identity, minority stress, and mental health and wellbeing (Adler, 2012; Fingerhut et al., 2010), though this theorizing has not been applied to SOGIECE. Given the growing evidence base about the harmful social and mental health impacts of SOGEICE (Goodyear et al., 2022), there is need to understand the processes surrounding SOGIECE that impact identity to better address the mental health needs of the population.

## Methodology

### Master Narratives Framework

Master narratives framework encourages us to consider structural-psychological relationships and processes that individuals engage with as they develop personal narratives throughout their lives (Hammack, 2008; McLean & Syed, 2015). It is useful to understand the unique situatedness of marginalized groups as it further contextualizes individuals’ experiences and the social structures that shape their lives (Syed & McLean, 2021).

Identity is shaped by the social contexts we grow up in, namely, master narratives (Hammack, 2008). Master narratives are shared stories of how dominant society makes sense of the world (Boje, 1991; McLean & Syed, 2015) which teach us from early childhood how to behave and shape our lives according to expectations of social acceptability – how to aspire to the human ideal within a given cultural context. They are often produced and sustained by those in positions of power (Thorne & McLean, 2003). Personal narratives are the narratives that individuals construct for themselves (McLean & Syed, 2015). People whose personal narratives function in tandem with societal master narratives are often unaware of these master narratives – their lives benefit from them (Syed & McLean, 2021; Thorne & McLean, 2003). SGM people, however, construct their narratives amidst master narratives that are frequently in opposition to their personal narratives, where the master narrative is one of cisheteronormative identities and behaviours. For those whose personal narratives are at odds with the cultural master narratives, such as those with minority identities, they "…may need to construct or adopt an *alternative* narrative, which at minimum differs from, and at maximum resists, a master narrative" (McLean & Syed, 2015, p. 320).

The objective of this article was to understand identity, as a life story, by examining the processes between individuals (personal narratives) and structures (master narratives) among SGM who have experienced SOGIECE. We used the master narrative framework to focus on the individual-structural interplay and describe master and personal narratives with particular attention to the processes that SGM who have experienced SOGIECE engage with as they navigate master narratives reinforced by SOGIECE (Syed & McLean, 2021). By doing so, we might encourage health professionals to deemphasize cisheteronormativity within the systems in which they work and prompt SGM researchers to consider the structural contexts that stifle SGM people’s lives and impact their health.

### Theory

Studying master narratives inherently centres the operation of power and inequity in society (Syed & McLean, 2021). We approach this study from a critical social paradigm (Eakin et al., 1996) to uncover and expose how power structures operate in the lives of SGM people who have experienced SOGIECE. Critical social theory, particularly queer theory, situates us to be attuned to particular social structures that reproduce cisheteronormativity and perpetuate harm towards SGM people (FitzGerald & Rayter, 2012). This work is intended to question the status quo and promote social justice.

### Reflexivity and Positionality

The construction of this inquiry derived from reactions throughout data collection and analysis by the first author, who became attuned to the structural forces that participants’ stories seemed to exist within and the impact these forces had on participants’ identities and belonging. This inquiry is shaped by the authors’ experiences as queer men whose identities were strongly impacted by SOGIECE; our lives mirror many of the stories told by participants and motivates our work (Kinitz, 2020; Salway, 2020). We believe that sexual orientations and gender identities that do not align with heterosexual and/or cisgender norms are valid, equal, and require no intervention. Throughout data collection and analysis, the first author wrote reflexive memos observing emotional responses to participants, engaged in dialogue with colleagues to ensure the data analysis adhered to the essence of participants’ experiences, and responded to reflexive questions to understand how assumptions and views might impact analysis (Mauthner & Doucet, 2003).

### Narrative Inquiry

One participant, a cisgender gay man in his 40s who described being sick of having others reject his story, articulates why narrative inquiry is an appropriate methodology for this study.

*“I mean…it’s like this blatant ignorance of the harms that it’s caused and not willingness to listen to stories. It’s almost like it muffles people’s stories, or it takes your stories, like it rips your story from you and says ‘No, this isn’t your story,* ***this*** *is your story.’ And very much puts an identity and says, ‘You know, you’re not gay, you’re same-sex attracted, and you know, we can work on this and we can fix this.’”*

Those who have experienced SOGIECE have historically been silenced, and their stories need to be told. From analysis to representation, narrative inquiry is a creative analytical practice that centres on the lived experience of those whose stories are often silenced (Kim, 2015). Narrative permits us to garner meaning and understanding of people’s lives in a social context (Polkinghorn, 1995). Employing this methodology, we embrace complexity and ‘messiness’ of SOGIECE and identity development beyond the focus of the inquiry (i.e., SGM identity) by attending to the temporality, sociality, and spatiality of participants’ stories in broader sociocultural contexts (Kim, 2015).

### Data Generation

This article presents findings from a study among SGM across Canada who have experienced SOGIECE. We recruited participants with experiences of ‘conversion therapy’ using purposive sampling to maximize diversity, through advertisements distributed across Canadian community organizations’ (e.g., Community-Based Research Centre) listservs, and social media (Green & Thorogood, 2018). Targeted outreach through word-of-mouth and advertisements were shared with organizations who work closely with specific populations (e.g., trans, Two-Spirit and racialized communities) within SGM communities. Participants were included in the study if they were at least 19 years old, living in Canada, spoke English or French, and reported to have experienced ‘conversion therapy’. Study tools and protocols were reviewed and approved through Simon Fraser University’s Research Ethics January 9, 2020 (#2019s0394), and Université de Montréal’s Comité d’ethique de la recherche en sciences et en santé May 15, 2020 (#CERSES-20-048-D).

Between January and July 2020, three graduate student researchers, two men and one woman, with backgrounds in public health, social work, and nursing, conducted 22 semi-structured interviews lasting 60–150 min in length. Interviewers had personal connections to SGM communities and conducted this work in Canada’s three most populous cities: Toronto, Montreal, and Vancouver. Interviews were undertaken primarily virtually due to COVID-19 pandemic restrictions; four participants provided written consent and were conducted in person, and 18 provided verbal and were conducted virtually. The interviews opened narratively with a broad prompt to allow participants to share their experiences of SOGIECE (Kim, 2015): ‘Tell me your story about experiencing conversion therapy’. Following the initial storytelling from the participant, subsequent questions were asked about the participants’ relationships and ideas about the future, such as ‘What does involvement with LGBTQ2 communities look like in your life?’, ‘How has conversion therapy influenced your experiences with, or connection to, LGBTQ2 communities?’ and ‘Why does conversion therapy continue to happen?’ Participants were diverse and self-identified with respect to gender (17 men, four women and four non-binary/genderqueer), race (17 white, 1 Southeast Asian, 1 Black, 1 First Nations, 1 Arab and 1 multi-racial), age (six 19–29 year-olds, 11 30–49 year-olds and five 50-year-old and up), and sexual orientation (15 gay, five queer and four bisexual/pansexual). See Kinitz et al. (2022) for more detailed demographics. Interviews were transcribed verbatim and stripped of identifying information. NVivo 12 software was used to manage transcripts and analytical notes.

### Analysis

We drew on Pokinghorne’s (1995) narrative analysis, which prompts the analyst to engage in the ‘emplotment’ process, identifying a plot and narrative configuration as analytic tools. Narrative analysis allowed us to ensure the analytic process did not break apart the data but rather combine it in a way that made it cohesive and created meaning. The first author began data analysis by closely reading and re-reading interview transcripts to immerse in the events and experiences told by participants. Once deeply familiar with the data, the first author started to recognize and identify a plot that brought together aspects of participants’ interviews (e.g., events, situations, actions) and configured these elements chronologically (Polkinghorne, 1995).

Considering the plot and connections of participants’ experiences to understand master narratives, identity, belonging, and related concepts, elements of interviews were coded. Coding began deductively (e.g., identity, belonging, heterosexism) followed by more detailed, inductive coding (e.g., rejection, bullying). Codes and concepts that did not clearly align with the plot were analysed for contextual relation to the primary concepts identified through coding. We were attuned to master narratives that are entangled in power relations in the storied lives of SGM people; namely, we explicitly looked for master narratives of cisheteronormativity and other structural forces that impact the identities of SGM people. This required an iterative engagement with and re-reading of the transcripts and codes to understand the master narratives outside the concepts of focus (i.e., identity, development and belonging; Polkinghorne, 1995). Throughout the analysis, we considered and interrogated the relationships between various concepts, plotlines and master narratives. Further analytic strategies were employed following Eakin & Gladstone's "value adding" analysis, such as reading the data for the gestalt, where the first author reviewed full transcripts for the broader context, or “bigger happenings”, within the data (Eakin & Gladstone, 2020). This was done both prior to and following coding to ensure that our coding of the data did not limit our interpretations.

### Representation

Results are presented as a single story based on a holistic understanding of what participants shared in interviews. We draw from Bildungsroman, a form of biographical narrative inquiry, to focus on the story of participants’ developmental journey and the resistance or acceptance of master narratives to foster a sense of self (Kim & Zimmerman, 2017). Bildung is a philosophical concept that centres how people develop their sense of self through life trials (Gadamer, 2013; Kim & Zimmerman, 2017). Creative non-fiction helps us to illuminate the tensions, conflicts, and journeys of participants’ experiences by using expressive language and creative writing techniques to generate an illustration of the reality of participants and evoke empathy (Gutkind, 2012). We use metaphor, conversation, and imaginary characters, rather than trying to simply retell the ‘facts’ of participants’ stories through reductionist means (Caulley, 2008), hoping that participants voices not just be heard but *felt*. Constant comparison between the creative non-fiction and the analysis was critical to stay true to the findings and stories of participants (Caulley, 2008; Kim, 2015). For illustrative quotes, please see Appendix A.

## Results

### Sam’s Story: a Creative Non-Fiction

#### Chapter 1: Learning the Master Narrative: Heterosexism Rules All 

"Yo, faggot!" Sam heard them yell from across the street. It was one of the guys from school – one of the many guys from school who had harassed and assaulted Sam in the science lab the day before. He was skipping the afternoon periods today to avoid another elbow to the back during lunch or an after-school run-in leading to more bruised ribs. "You going to go cry to your boyfriend, fag?" the guy yelled again. Sam could not walk any faster without drawing more attention in, what he referred to as, "this fucking hick town".

All his life, Sam heard his mom say, "God has a plan for your life, Sam."; "God doesn’t make mistakes, Sam." Sam knew God had made a mistake when it came to his life though. Sam grew up going to church, memorizing the Bible, being a leader at the church’s summer camp and maintaining a strong connection with his parents. He fervently desired to make his parents proud. He believed in God and wanted to follow the path he was taught by his mom and dad, teachers, and community – the path he was expected to adopt as his own: a family, with a wife and kids he could be proud to bring home on the holidays; a job as a schoolteacher, sharing his passion for music and educating the next generation to be better than the last.

Holding tighter and tighter to these dreams, Sam felt different from others at his school. Sex-ed class was awkward with his friends cracking jokes. He never understood what they were talking about when they would comment about being attracted to someone. He did not see characters on TV and think about them as sexual beings. Sam would not allow himself to go there, to understand what it might be like to feel attracted to another person.

Going through puberty, his creative mind would transport him back to the lively sermons where bubbling saliva would fly out of the preacher’s mouth, so passionately expressing the dangers of the world. "The devil is trying to tempt us with worldly pleasures; homosexuality, bestiality, pedophilia – a slippery slope that earns you a one-way ticket to hell," he would recall. The further into puberty Sam went, the more confusing his life became. His feelings grew stronger, away from the singular attraction he was taught to have towards women. Like a mountain climber gripping to a ledge during a gale of wind, he clung harder to his desire to only like women, to make his family proud, and stay away from feelings that would surely lead to paedophilia and a life of eternal damnation.

Sam lay face down on his bed weeping. This was not how 15 was supposed to be. His dreams on pause, not knowing how to move forward. He cried himself to sleep most nights that year, feeling confused, knowing something was wrong, and begging for God to make these feelings go away. Alas, Sam woke up each morning, tired and tense. Isolated, and feeling underserving of love and connection, Sam’s self-esteem was like a flag at half-mast on a still day. He swore to himself that these feelings would be his most closely held secret, desperate to find a way to rid himself of the disease that was taking hold of his body, the illness that kept him on the outside, desiring to be normal. His attraction to men was his Sisyphus trial, taking all his effort but achieving nothing.

#### Chapter 2: If It Doesn’t Fit, You’re Broken: Internalizing the Master Narrative

Sam went to the pastor at the church his family went to to ask how to get rid of his unwanted feelings of attraction towards men. His pastor gave him the number of someone in a neighbouring church who had experience working with people who had similar struggles; "a counsellor of sorts" the pastor said.

"I’m so glad you’re here, Sam. I’m sure we will be able to find out why you are feeling the way you are. Sometimes it’s because your mom was too much of an influence or your dad wasn’t around enough. What is your relationship like with your parents, Sam?" Sam’s counsellor, Allan, questioned.

The smell of the office was a familiar one, having grown up in the church; the dampness in the old church carpets and wooden walls being blown around by a swinging ceiling-fan. "My parents are the reason for this? I think my parents are pretty normal. I get along with them fine. I was always the favourite", Sam claimed.

“Were you ever left alone with an adult as a child, where someone might have touched or molested you?” The questions continued. "When you’re watching TV or movies, do you find you’re more attuned to characters of the same sex as you?" 

"Huh, I never thought about it, but I guess so?" Sam responded quietly, wracking his brain trying to place the blame somewhere. "I still find women attractive, so I guess I find myself interested in any characters, regardless of gender."

"For homework this week, I’m going to ask that you notice how you speak and some of your mannerisms that are not typical for a man. Try joining a sports team at school. It will help you better understand more masculine traits. And maybe cut down on the time you spend with some of your female friends." Sam reflected on what he was being taught in his meetings with Allan, questioning, processing, and beginning to internalize that one’s sexuality was moulded by gendered activities he had once sat out of or that he must have done something wrong to be burdened with this ‘same-sex attraction’. Allan’s questions trying to ‘connect-the-dots’ as to why Sam felt this way had Sam’s mind racing. He realized all the events of his past had made him who he was.

Months went by. Sam and Allan relentlessly pushed to find out the source of his attraction towards men. Sam questioned his relationships, resisting blame towards his parents that was explained by Allan as a source for same-sex attraction. Sam had a great relationship with his parents though. "Why was Allan trying to malign this one positive source of support?" Sam questioned defensively but with doubt sneaking in that Allan’s argumentation might have some merit. Sam felt he just needed to try harder and interrogate every aspect of his being to figure out the cause of his brokenness. He learned that the blame lay within and could feel the burning heat of eternal damnation as if he were an ant trapped by a kid with a magnifying glass at high noon.

Sam invested hours each week learning how to ‘bounce his eyes’ when looking at a man. He learned to act more masculine, to pinpoint what in his life resulted in his immoral desires, or to explore many other strategies to suppress any feelings of desire, emotion, or love towards men. He devoted everything to avoid the parts of himself that were not in line with what he wanted for his life, or at least what he was taught to want for his life. Every day, he would pray that God fix him, resist any feelings of attraction towards men, and practice the masculine tendencies he was taught would help heal him. This went on for years. "Had it been years?" Sam reflected, trying to detail the various components of programming he had endured, each blending into the next.

Sam learned to be terrified of new acquaintances who might validate the expressions of love and care that he was trying so hard to suppress. Symbols of SGM communities became symbols to dread and detest, representing what Sam was adamantly resisting. Ultimately, Sam spent two decades of his life not willing to consider that being attracted to men was okay – he was taught to fear and hate himself.

There were days where Sam considered giving up. He would skip the odd appointment with Allan, but the self-loathing and shame would eat at him, leading him back to Allan’s promises of change. The bullying at school continued, where Sam was physically and verbally assaulted. He always felt like the odd one out seeing straight couples at the mall, at church, and on TV live out their lives freely. Sam felt like the black sheep of his family, school, and anywhere he went. With each interaction, he was reminded that he was different – broken.

"You need to pray harder", Allan would encourage Sam. "It is going to be a battle for the rest of your life, but you need to stay committed."

"I can’t do this anymore!" Sam sobbed to Allan. "I’m a failure. Why can’t I change? Nothing is working. I’m trying so fucking hard." Sam sat in Allan’s office, having never felt so lost, so broken, so empty. Allan was taken back for a moment, but this was not the first time Allan worked with someone like Sam. Allan assured Sam, once again, that this was going to be a journey to struggle through for the rest of his life, assuring that it would continue to get easier.

#### Chapter 3: Searching for Alternatives: The Quest for a New Narrative

Sam’s relationship to queer communities was fraught with fear, curiosity, and shame. He described his relationship through the metaphor of a queer party that he was not sure about and rejected when invitations were offered:

Sam sat on the edge of a cool, damp planter that lined a dimly lit parking lot. On the other side of the street, Sam could see the makings of a party: bright lights and flashes of colour, the sounds of laughter and music. He could feel the energy of happiness, an energy he had been without for too long. He wanted to cross over from this desolate and empty lot but could not. He felt the weight of a lifetime of shame holding him back from feeling he could join the party across the street. Allan’s voice, the voice of his high school bullies, his parents, all filled Sam’s head. "You’ll always feel an emptiness if you enter that life—the homosexual life."

Sam sat on the edge of that planter, thinking, "What is the gay ‘lifestyle’, anyhow? I’ve resisted any familiarity with it for so long. It’s a lifestyle of sin, filled with promiscuity, emptiness, and insatiable desire. I could never feel comfortable going over there, pretending that I belong in *that* community."

It had been 8 years since Sam first went to his pastor, and 4 years since the last time he saw Allan. And yet, Sam was there, sitting on the planter, gazing longingly at the party across the street he had denied from himself. He yearned for happiness, connection, and intimacy, but the fear was still all consuming.

“Hey, what are you doing over here?” a peppy-voiced man with short shorts called to Sam as he jogged across the street.

“Hi. Nothing, just thinking.” Sam mumbled nervously, not wanting to look too engaged. “I’m okay. Don’t worry about me.”

“I was just taking a breather and thought I’d come say hi. You look lonely over here. You’re welcome to come join us if you want.”

Sam rejected the offer, scared of getting close to anyone, scared of validating that part of himself that he has spent years trying to exterminate. He received many welcomes to the queer community, just like the peppy-voiced man from the party, but he felt safer in the solitude of the dimly lit parking lot. The friends Sam managed to make quickly jumped ship after hearing Sam’s desire to be straight and the shaming rhetoric he would spew at the first sign of sexual expression. Sam felt stuck, being pulled by his family and community to live a faithful, heterosexual life, while new acquaintances he had met from university and online urged him to forget about his religious roots and to embrace his authentic, bisexual identity.

Sam went back to the metaphorical planter every so often, trying to figure out what he wanted in life. No one, not even Sam, had ever paused to ask what *he* wanted before. The following years were filled with new experiences followed by confusion, shame, and guilt that harkened back to the demonizing of same-gender attraction Sam experienced. Sam tried to date guys – which ranged from abandonment at the sign of intense internalized homophobia to breaking down in tears after a loving embrace from his new boyfriend – and make new friends, do the mental gymnastics of trying to figure out faith and sexuality, and reconfigure his shattered identity.

#### Chapter 4: Adopting an Alternative Narrative: Embracing Self-Love Amidst the Pain

Sam woke up startled. "Wake up!" his ostentatious roommate hollered. "We’re going to the market – wanna come?" Sam stretched as his eyes adjusted, and a smile came over his face. "I can’t", Sam called back. "I have the queer men’s chorus at noon. I don’t want to miss practice."

Now with the words to label what it was that he went through – conversion therapy – Sam was able to process and admit to himself that these experiences were not okay. He had spent the past 5 years in an affirming therapist’s office working to deconstruct his internalized shame and rebuild his fragmented identity. Sam had learned from his therapist how to manage the angst of holding sexuality alongside his faith. He had learned to be less judgemental toward himself. He had learned to love himself.

Sam has put some distance between himself and his family and conservative church. He is now a teacher, has friends whom he loves, and has had positive experiences dating. However, he continues to manage the intrusive thoughts. He worries his fellow teachers will think he is a predator, he feels gut-wrenching guilt after sex, and has doubts that he can really be happy living in this alternative narrative: proud and bisexual. He is reminded of values he was raised with every time he goes home for a holiday or encounters people who hold restrictive views on love and relationships.

Finding he has the house to himself after choir practice, Sam lay on his bed, thinking: "It has been a 20-year journey, but I’m finally happy. I wish it didn’t take me so long, that I didn’t think being attracted to men was something to be afraid of. But I’m so glad I’m here. I’m ready to start again, to experience and learn what I was supposed to 20 years ago: what relationship is best for me? How can I tell if I’m attracted to someone? How can I ensure I feel I’m worth loving? What do *I* want? I’m free to make my own choices for the first time in my life. I no longer feel broken, I’m just excited for the future."

*∼* ***NOT*** *the End ∼*

## Discussion

We show that SOGIECE are not isolated experiences in which SGM learn social expectations to be straight or cisgender, but rather that they are constituted by the internalization of cisheteronormative master narratives that accumulate throughout childhood, leading to a desire to align with heterosexual expectations. Those with experiences of SOGIECE often spend years actively trying to reject SGM identities to align with cisheteronormative master narratives, even before enrolling in any formal SOGIECE programming. Nonetheless, those who experience SOGIECE are often able to adopt an alternative narrative aligned with a SGM affirming stance; though, many continue to navigate feelings of brokenness years after SOGIECE due to the internalized master narratives asserted in their formative years. Our analysis illuminates processes of internalization, self-loathing, and unlearning with which SGM who have experienced SOGIECE engage in order to construct a personal narrative amidst cisheteronormative master narratives (Hammack, 2006). These structures and processes thwart SGM identities leading to delays in adopting alternative narratives. Such rich stories add contextual nuance to much of the quantitative SOGIECE literature that highlights the relationship between SOGIECE and deleterious mental health outcomes. This nuance might assist healthcare providers and researchers to better understand areas of intervention for those who experience SOGIECE.

People make meaning of their lives through the construction of personal narratives (Cohler, 1982). However, SOGIECE prohibits adoption of personal narratives that do not align with master narratives and disparages and pathologizes aspects of one’s identity, thereby impacting and delaying SGM’s positive/affirming identity development (Hammack et al., 2013; Henry, 2013). Participants from this study were from diverse cultural backgrounds and religions that disparaged sexual or gender diversity, leading participants to SOGIECE. Growing up in anti-SGM settings and/or learning about SGM people as deviant lead to internalized homonegativity, a known predictor of attending SOGIECE (Tozer, 2001). Once in SOGIECE contexts, individuals learn to internalize and be silenced by master cultural narratives that restrict personal narrative construction that shape identity (Syed & McLean, 2021). This is articulated by prominent proponents of SOGIECE: “…approaches have in common the goal of attempting to help dissatisfied homosexually-oriented people learn to resist and minimize their homosexual behaviours, thoughts, and feelings so that they can live more happily within the mainstream heterosexual culture which they value” (Dean Byrd et al., 2008, p. 3). The years and active energy resisting SGM outcomes or identities in everyday life and amplified through SOGIECE left many participants experiencing significant delay in adopting an alternative narrative and secure identity. Due to the silencing of narratives that resist cisheteronomative master narratives, participants had to navigate important aspects of their identity later in life, such as faith and sexuality, sexuality and gender, love and romance, and feelings of self-acceptance.

We can draw on Cooley's 1902 theorization of ‘looking-glass self’ to consider how master narratives framework might materialize through the everyday operations of power (O’brien, 2006). The looking-glass self theory posits that our social interactions inform our identities, such that we define our social identities based on others’ perceptions of us (O’brien, 2006). Those who have experienced SOGIECE often have been informed by family, peers, community, and broader society that they are deviant, broken, and need to be corrected (Kinitz et al., 2022; UK Government Equities Office, 2021), which then impacts their sense of self and affirms the perspectives of their social networks. In master narratives framework, Syed & McLean (2021) explain that "structural systems that uphold White supremacy, patriarchy, heteronormativity, and Christianity, to name a few, are not just “contexts” in which individuals develop, but rather are part of the fabric of their everyday lives" (p. 2). Environmental and social contexts in which SGM people exist play a significant role in identity development (Diamond & Savin-Williams, 2003). These contexts can lead SGM people to SOGIECE, such as unsupportive families and communities that foster beliefs that SGM people must change, as well as support them in their experiences post-SOGIECE, such as finding accepting and affirming spaces and communities that are essential for identity development and wellbeing (Ryan et al., 2020).

The ability to construct an alternative narrative to dominant cisheteronormative narratives can take years for SGM, consisting of stories of struggle and strength (Hammack, 2018). Participants in our study felt that to release the grip of cisheteronormative master narratives and adopt an alternative narrative, they often needed to cut ties with or distance from family and communities of origin who perceived them as deviant and radically reject the master narratives they internalized throughout their lives. Others continue to navigate master narratives, some more or less adopting aspects of cisheteronormativity to align with the master narratives available to them. To bolster identity development and wellbeing, participants turn to SGM communities for support and belonging; communities where they are perceived as normal. After years of feeling isolated from their community of origin while simultaneously rejecting, or feeling rejected, from SGM communities, those who have experienced SOGIECE are able to strengthen their new personal narratives. This was done when surrounded by supportive and accepting communities, such as the queer choir in the story of Sam. Though often rife with internalized oppressive narratives, SGM identity development and wellbeing were bolstered with community support and feelings of belonging (Barr et al., 2016). Sam’s story illuminates that irrespective of where participants were in their identity journey, negotiation of sexual orientation, gender identity, and/or gender expression was an ongoing task. Participants shared stories of extreme adversity and strength as they continued to manage the lasting impacts of SOGIECE on their adoption of alternative narratives, identities, social life, and mental health.

The anguish that the protagonist endured navigating oppressive master narratives resulted in many years of struggle and delay of adopting a personal narrative that forms his identity. However, the adoption of alternative narratives that resist master narratives are part of a social process that has potential for disruption and social transformation (Fish & Counts, 2020; Stetsenko & Arievitch, 2004). The processes that are experienced by SGM who experience these master narratives through amplified means, such as SOGIECE, require either adjusting to social expectations of straight or cisgender identities or engaging in active work to adjust to an alternative narrative (Cohler & Hammack, 2007). As more SGM engage in adopting alternative narratives, which are both fostered and constrained by social and material conditions (Hammack, 2006), it is possible that the journeys of identity development for SGM will improve. Interventions are needed that make it easier for SGM to engage with their social structural realities that permit them to sooner and more easily adopt personal narratives that shape their identities and improve their mental health. If we are to understand master narratives as cultural products, then we as health professionals and researchers must make changes in our everyday lives, and the policies and practices that structure our lives, to shift culture and subsequent master narratives that stifle the healthy development of SGM.

### Practice Implications

For those in medicine, social work, psychology, and other healthcare professions, we wish to highlight the operation of oppressive structures in the lives of SGM. Given the mental health concerns for SGM who have experienced SOGIECE, healthcare professions will undoubtably work with those whose narratives reflect those in the story above. In our practices, there is a need to go beyond strengthening resilience and move toward dismantling the systems that require SGM to be resilient in the first place. Active efforts to confront and challenge the operation of cisheterosexism in schools, media, public policy, religious institutions, as well as from within caring professions, are needed. These could include trauma-informed education for healthcare and caring professions, identifying and supporting institutional (education system, religious organizations, workplaces) practices that embrace gender and sexual diversity, or sharing of both harms and successes of those in SGM communities through mainstream outlets (Ending conversion therapy in Canada: Survivors, community leaders, researchers, andallies address the current and future states of sexual orientation and gender identity and expression change efforts, 2020)\x{200b}. Further, clinicians and service providers might draw insights from this study to provide trauma-informed mental health care related to grief and loss, supporting religious desires alongside sexual and gender diversity, and reducing shame and stigma while strengthening social supports (Horner, 2010; Jones et al., 2022; Power et al., 2022).

### Limitations and Next Steps

Results are largely influenced by perspectives of cisgender men who have experienced SOGIECE through Western faiths (e.g., Chritianity), and Sam’s story and specifics of the master narratives analysed may not apply across the diversities of SGM identities. Further, this story is particular to the context of North America, where the vast majority of people characterize conversion practices as harmful and where SGM rights are enshrined in law; we acknowledge, however, that this limits the transferability of the results to other places where majority viewpoints continue to stigmatize SGM. Previous studies have identified negative mental health impacts of SOGIECE (Salway et al., 2020; Turban et al., 2019) with little exploration of the individual-structural processes, such as constructing identity amidst amplified cisheteronormativity, that might contribute to poorer mental health. We suggest that participants’ stories, as represented above, highlight the impacts of SOGIECE on their identity development that then resulted in years of confusion, self-loathing, and mental health challenges. Cultural ideologies, such as cisheteronormativity, severely constrain human development (Hammack et al., 2013), particularly when these oppressive ideologies are amplified through targeted and sustained practices, such as SOGIECE. We propose that future studies should therefore test the pathway between SOGIECE and mental health to evaluate the importance of identity constraint as a possible mediator of the effects of SOGIECE on mental health struggles among SGM.

## Conclusion

Our analysis provides insight into the fallout of SOGIECE among SGM and highlights the impact of emboldened cisheteronormativity on SGM identity. We build upon literature focussed on the measurable harms associated with SOGIECE (e.g., suicide) by drawing attention to some of the more hidden, or inner, harms that are impactful to health and wellbeing. This study, and others that assess the harms of SOGIECE (Jones et al., 2021), provide further evidence that SOGIECE is an unethical practice for many reasons but most notably the harms associated with identity and mental health. Knowledgeable and accessible mental health and psychotherapeutic supports are needed for SGM who have experienced SOGIECE. Health professions need to consider and dismantle the active presence and lasting impacts of master narratives, such as cisheteronormativity, when working with those who have experienced SOGIECE, as well as SGM communities more generally.

## Appendix A Illustrative Quotes

### Illustrative Participant Quotes for Chapter 1:

“*I felt very, very much alone, I felt weird, different, I felt well in the, sorry I keep referring back to the bible, I was so programmed at the time, but there’s, when there are stories in the bible that talk about leprosy I always identified with those characters that had leprosy because they were left out on the fringe of town, they weren’t allowed to touch anyone, they weren’t allowed to get close to anyone and if they did go into town they had to announce that they were unclean the whole time that they were there, and I always felt that that was a powerful metaphor for how LGBT folks you know feel in a church setting. It was definitely how I felt the whole time I grew up that way….when I was a kid I mean I got the shit beat out of me and called a fag many times… I had that feeling of shame as if I was a leper always.” – 40-45-year-old, cisgender bisexual man*

*“I remember just having this kind of internal struggle about what I was feeling and my identity and this attraction to women and…I’ve got this burden and I wanted to get healing, like I wanted this not to be the reality, something’s wrong with me so it needs to be fixed.” – 40-45-year-old, cisgender queer woman*

*“I think back to my time in a rural congregation that had no awareness, like a rural town, a rural congregation that really had no awareness of like diverging in sexuality, the diversity in sexuality or gender expression, gender identity…especially in a rural context because there’s not a lot of, at that time anyway there wasn’t a lot of, there wasn’t like a P-flag or that type of thing in town, there was no yah there was just not supports. I was not aware of any other gay people in my town or in my high school even.” – 40-45-year-old, cisgender gay man*

### Illustrative Participant Quotes for Chapter 2:

*“…like you don’t leave conversion therapy, like your one-hour session. I would go back to high school, continue with classes and that…but I wanted that so bad from the counselling sessions that that is involved in your entire every day as well, like 24/7 you’re praying with your parents before you go to bed that they’ll pray the gay away kind of thing and that god will change you and then you’re crying yourself to sleep praying that god will change you and those kinds of things cause they were like you just have to try harder. And you can’t, like there’s no extent that you can try that’s ever going to work so it’s just like try, try, try but you’ll never be successful and that kind of really sinks in after a while. So yah during that time it was a rough go cause I felt like I was trying…” – 25-29-year-old, cisgender queer man*

*“…be caught in this endless cycle. Never once crossed my mind that maybe it was okay for me to be bi. And, but it just like it just ground deeper and deeper into me this sense that I’m broken, that I’m incomplete. And so conversations around homosexuality and meetings where we’d talk about or pray through what could be the root causes of that in my life you know we did have those kinds of meetings.” – 40-45-year-old, cisgender bisexual man*

*“They made me feel like I was failing, they made me feel like I’m not trying hard enough because to me it all made sense and I just had to kind of apply myself to this program but it wasn’t working and so it was kind of like my last little glimmer of hope was dissipating and I had nothing else to rely on and I was just going to be super unhappy for the rest of my life because I couldn’t do this. So, I kind of took it as a personal failure. Eventually that led to a very dark period in my life where I was suicidal and just things were not going well…So, I was very, very involved, I really wanted it to work and I think that’s why it was so damaging for me, because I put everything I had into it. And I put all my hope and my belief into it, and moving away from that was also very difficult, because the shame and the guilt lingered for a very long time.” 25-29-year-old queer non-binary, trans person*

### Illustrative Participant Quotes for Chapter 3:

*“So, you can be like out of conversion therapy, but then it takes a long time to get out of purity culture and get out of the idea, like understand it’s okay to have sex. I’m like slowly wrapping my head around this, I’m [40’s], I should have figured this out at 16 which is the average age, 17 would be losing your virginity. So, you lose like 35 years of your life basically. And that really sucks, ‘cause you can’t get that back and developmentally you’re just delayed, very like yah so that’s really painful. So, in terms of like developing relationships, it’s totally like, I think it’s really messed it up cause I can’t tell what’s happening inside of me…“How can you tell if you’re attracted to somebody?”. That’s like something people should just know, that’s an honest question how can I tell if I’m attracted to somebody…So even trying to figure out what it is that I want. What do I actually want, that’s not a way that I’ve ever lived, cause it was what does everybody else want…” – 40-45-year-old, cisgender gay man*

*“I guess the years I just spent in fear and not pursuing relationships. I remember having this vivid idea of, I guess as this person who was very in the closet, I felt like I was standing at a door, there was a window in the door, there was this party going on, you know this LGBTQ party and I’m just standing there at this door looking in and terrified to enter and you know there’s this party happening and I kind of want to go in but I’m terrified, and if I go in it’s a slippery slope and am I on my way to hell in a handbasket kind of a thing so. All those years I spent at that door looking in and wanting, really wanting intimacy and wanting to make really close, you know just I think we all need intimacy at some level…So, just all those years spent trying to fit into the church and denying myself the opportunity to express myself sexually or to allow myself to enjoy having a crush on somebody or to think that person is really attractive you know. Instead of all the shame that compounded on me. So, I guess, yah all those years I’ve wasted were quite a big, that was part of the harm of SOGIECE was just keeping me in the closet and keeping me.” – 40-45-year-old, cisgender gay man*

### Illustrative Participant Quotes for Chapter 4:

*“And like I say I don’t, it’s only maybe in the past year where I’m kind of like so is the LGBTQ my tribe now and kind of like I don’t know, maybe…Yah but I’m kind of like you know what I kind of enjoy, I’m enjoying the camaraderie that I have now, I feel comfortable enough in the community that I feel like I can be a part of it. And that’s, like so it’s been a twenty-year journey to come to that level of comfort but I’m so glad I got here. And I wish like, I wish I had understood that it wasn’t something I had to be afraid of, you know, when I was younger but I had no way to know. I was programmed in something else.” – 40-45-year-old, cisgender bisexual man*

*“I feel free to make my own choices for the first time in my life. My life is now half over, but for the first time in my life, I feel free to do that, so yah…I feel like I’ve just moved on to a better stage in my life and ultimately a community that’s a better fit for me.” – 40-45-year-old, cisgender bisexual man*

*“I would say first of all I’ve, well I still identify as a Christian, I am, I struggle with being a part of the traditional Christian community, that’s something I do, I don’t, yah I do struggle. I do like to attend more liberal congregations and I, it’s something that I would eventually like to get back into. It hasn’t really happened yet, so giving myself space I guess, space and distance just from, I feel a lot of my issues are with the church in general or sort of with the experience of rejection, of shame and all that so it’s been part of a, being part of queer faith community, having SOGIECE experiences is, you know there are other people who have experienced it so you know sharing experiences, talking about it, hearing how other people are processing and interpreting their faith have been really helpful. I think also it was, coming out was a very slow, gradual, painful process and you know being able to finally come out to my parents when I was in my 30s and sort of they were very understanding and have been very supportive so. . . I think also having you know being connected with the people who through [name of affirming ministry], through the queer faith community, knowing that I guess you know seeing other people live with a balance. Being able to have their sexuality and their faith and you know figuring that out together and not being, not having shame around your sexuality, seeing people who lived openly and out and having you know having partners, and being also people who identify as Christian.” – 40-45-year-old, cisgender gay man*
